# Properties of ZnO Prepared by Polymeric Citrate Amorphous Precursor Method: Influence of Cobalt Concentration

**DOI:** 10.3390/ma18173991

**Published:** 2025-08-26

**Authors:** Jailes J. Beltrán, Luis A. Flórez, Luis C. Sánchez

**Affiliations:** 1 Departamento de Química, Universidad de Córdoba, Cra. 6 #77-305, Montería 230001, Colombia; lflorezgalvan48@correo.unicordoba.edu.co; 2 Departamento de Física, Universidad de Córdoba, Cra. 6 #77-305, Montería 230001, Colombia; luiscarlos@correo.unicordoba.edu.co

**Keywords:** TGA-FTIR, XRD, FTIR, SEM, magnetic properties, room temperature ferromagnetism

## Abstract

This study aims to investigate the vibrational, structural, morphological, optical, and magnetic properties of Zn_1−x_Co_x_O with 0.00 ≤ x ≤ 0.05 prepared by the sol–gel method via an amorphous citrate precursor. FTIR spectroscopy was used to follow the thermal decomposition process of the ZnO precursor, identifying acetate zinc as the intermediate main component. XRD and FTIR-ATR techniques showed only the single wurtzite crystalline phase with the presence of oxygen deficiency and/or vacancies, and secondary phases were not detected. SEM micrographs showed agglomerated particles of irregular shape and size with a high distribution and evidenced particles of nanometric size with a morphology change for x = 0.05. We detected high–spin Co^2+^ ions located in the tetrahedral core and pseudo–octahedral surface sites, substituting Zn^2+^ ions. The energy band gap of the ZnO semiconductor decreased gradually when the Co doping concentration was increased. M vs. H for undoped ZnO nanoparticles exhibited a diamagnetic signal overlapped with a weak ferromagnetic signal at room temperature. Interestingly, temperature-dependent magnetization showed superparamagnetic behavior with a blocked state in the low temperature range. The Co–doped ZnO samples evidenced a weak ferromagnetic signal and a paramagnetic component, which increased with x. The saturation magnetization increased until x = 0.03 and then decreased for x = 0.05, while the coercive field gradually decreased.

## 1. Introduction

Studies on the physical and chemical properties of a wide range of nanometric systems currently occupy one of the most important areas of investigation in both basic and applied science. Variations in the composition of these materials can modify and/or combine more than one property, opening the door to new and interesting lines of research in the development of nanomaterials with a great variety of potential applications. Semiconductor oxides such as ZnO, TiO_2_, SnO_2_, WO_3_ In_2_O_3_, and CuO, among others, have received a lot of attention from researchers because of the execution of many novel applications [1]. ZnO is highlighted for exhibiting a broad band gap (3.37 eV) and a large exciton binding energy (60 meV). It has good transparency, high electron mobility, and high thermal and mechanical stability at room temperature (RT). Additionally, it has non–toxic and non–hazardous properties. Additionally, ZnO is a promising material due to its unique optical and electrical properties, which make it suitable for applications including optoelectronic and photonics devices, photocatalysts, solar cells, supercapacitors, fingerprint technologies, gas sensors, water treatment, energy storage, dye-sensitized solar cells, biomedical sciences (antibacterial, antioxidant, and drug delivery), etc. [1,2,3,4,5]. These potential applications can be controlled and improved in ZnO when small percentages of impurities are introduced into its crystalline structure. Among these dopants, metal transition (MT) and rare earth dopants are ideal candidates to achieve RT ferromagnetism to create a new generation of devices based on spintronics [6]. MT elements have exhibited higher ferromagnetic (FM) properties, in particular cobalt–doped ZnO, with high magnetization saturation (Ms) at RT and improved optical properties making it a suitable material in this field [7]. It is well known that the physical and chemical properties of Co–doped ZnO nanoparticles strongly depend on the choice of the chemical synthesis method, starting reactants, and conditions during sample preparation. Different chemical methods have been used to synthesize Co–doped ZnO powder, such as co–precipitation [8,9], the hydrothermal method [10], green synthesis [11,12], the sonochemical method [13], the microwave-assisted method [14], sol–gel [15], sol–gel auto–combustion [16,17], modified Pechini sol–gel [18], the amorphous citrate route, etc. [19]. The sol–gel method based on the amorphous citrate route is a simple and fast method due to there not being too much handling in the process. In addition, the method is low–cost, environmentally friendly, and scalable. It offers control over the material composition, permits the mixing of chemicals at the atomic level, and yields nanopowders with a fairly narrow particle size distribution depending on the sintered final temperature [20].

This method includes the formation of polymeric citrates of the metal, which brings about oxides upon decomposition. It is expected that when these chelates are heated to form the polymer resin, the cations will be uniformly dispersed throughout. The thermal decomposition of this polymeric network has rarely been reported [21]. In order to identify the products of decomposition of the polymeric chelate, as well as the probable intermediates obtained during the synthesis process, it was considered suitable to study the thermal decomposition of the polymeric precursor at different temperatures. Additionally, to the best of our knowledge, there are no reports that deal with the magnetic properties of Co–doped ZnO samples prepared by the polymeric citrate amorphous precursor method.

This study is mainly focused on presenting a qualitative, quantitative, and careful investigation of the vibrational, morphological, crystallographic, optical, and magnetic properties of Zn_1−x_Co_x_O with (0.00 ≤ x ≤ 0.05). This paper describes the formation of the compounds yielded during the steps of the decomposition of the ZnO polymeric precursor. The variations in the studied properties as a function of Co concentration are discussed in detail.

## 2. Materials and Methods

### 2.1. Sample Preparation

Zn_1−x_Co_x_O (0.00 ≤ x ≤ 0.05) nanopowders, where x represents the nominal molar concentration, were prepared by the sol–gel method via an amorphous citrate precursor. In a 250 mL beaker, required amounts (to theoretically obtain 1 g of sample) of Zn(NO_3_)_2_·6H_2_O (99%, Merck, Darmstadt, Germany), Co(NO_3_)_2_·6H_2_O (99.0%, Merck, Darmstadt, Germany), citric acid (CA) (99.5%, Merck, Darmstadt, Germany) monohydrate, with a molar ratio [(Zn + Co)/CA)] 1:3, and 100 mL of double distilled water and deionized were mixed for the preparation of the starting sol. The pH of the solution was not varied. The resultant sol was heated slowly on a hot plate at 80 °C under constant stirring. The solution was maintained at this temperature to evaporate the solvent and to promote polymerization until the stirring bar stopped by itself. In this last step, the solution became more viscous and finally turned into a wet gel. The wet gel thus formed was dried at 120 °C for 6 h, resulting in a solid mass, which is known as the polymeric precursor. Afterward, this powder was hand-ground in an agate mortar, sieved through a 150 μm sieve, and finally annealed in a tubular furnace at 450 °C (heating rate: 5 °C min) for 1 h.

### 2.2. Characterization Techniques

The thermal decomposition of the polymeric precursors was studied by thermogravimetric analysis (TGA) using a TA Instruments SDT Q600 (New Castle, DE, USA). The samples were analyzed at a heating rate of 10 °C/min in an air atmosphere from RT to 600 °C. Fourier transform infrared-attenuated total reflectance (FTIR-ATR) spectroscopy of the samples was recorded using a Thermo Scientific Nicolet iS5 (Madison, WI, USA) with an iD7 accessory using a diamond crystal with an experimental resolution of approximately 4 cm^−1^. A JEOL JSM 6490 scanning electron microscope (Mitaka, Tokyo, Japan) was utilized to obtain SEM images and EDS spectra. X-ray diffraction (XRD) patterns were collected at RT using an Aeris Malvern–PANalytical (Malvern, UK) with a Pixel 3D detector with a Cu–Kα source (λ = 1.5406 Å) in Bragg–Brentano geometry. The data were carried out in the range of 20–80 in 2θ with a step of 0.022° and step time of 29 s. The loose powder samples were leveled in the sample holder and mounted on a rotary disk to ensure a smooth surface and homogeneity in the data collected. The XRD patterns were fitted using the MAUD program (free version 2.99991) to obtain crystal lattice parameters and average crystallite size (Dv). RT optical absorption spectra in the ultraviolet and visible light wavelengths were measured using a UV–VIS Varian Cary 100 Bio Spectrophotometer (Walnut Creek, CA, USA) fitted with an integrating sphere diffuse reflectance accessory. Because the technique is sensitive to the sample amount, similar weights were used to carry out the analysis under the same conditions. The magnetic properties of the samples were studied using a PPMS (Physical Property Measurement System) Model 6000 (Quantum Design, San Diego, CA, USA) equipped with a superconducting magnet. The magnetic measurements were carried out as a function of the applied magnetic field (±0.5 T) at 300 K and as a function of temperature (10–300 K). The magnetic measurements were replicated and the results shown are an average of the data.

## 3. Results and Discussions

### 3.1. Thermogravimetric Analysis

**Figure 1:**
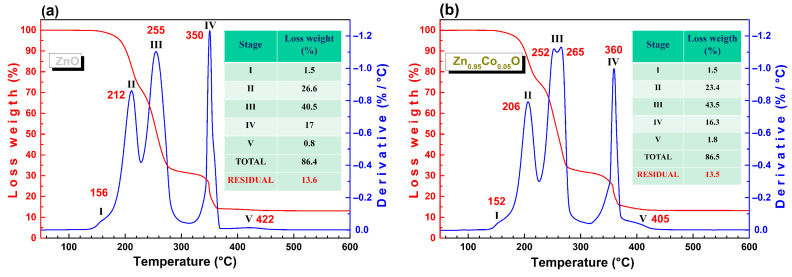
TG/DTG curves of polymeric precursors for (**a**) ZnO and (**b**) Zn_0.95_Co_0.05_O samples.

The first step in synthesizing ZnO nanoparticles by the sol–gel method via the citrate amorphous precursor process is the metallic chelate formation through the reaction between the zinc nitrate hexahydrate and CA. Because the Zn source is the limiting reactant, there is an excess of CA. During the gelation process (80 °C, slow evaporation of the solvent), a solution with high viscosity is formed, and NO_3_^−^ anions are eliminated as nitrogen oxide and molecular oxygen. After the drying process (120 °C), the gel is transformed into the polymeric precursor, very likely constituted by nonuniform polymeric chains. Figure 1 shows TGA/DTG curves of undoped ZnO and Zn_0.95_Co_0.05_O precursor powders, where DTG is the first derivative of the TGA. The different stages of the thermal decomposition process and their respective weight loss are shown in the Figure. For both curves, five similar regions of weight loss are observed in the thermal decomposition process. The thermal decomposition process of ZnO polymeric precursor was studied by FTIR spectroscopy.

**Figure 2:**
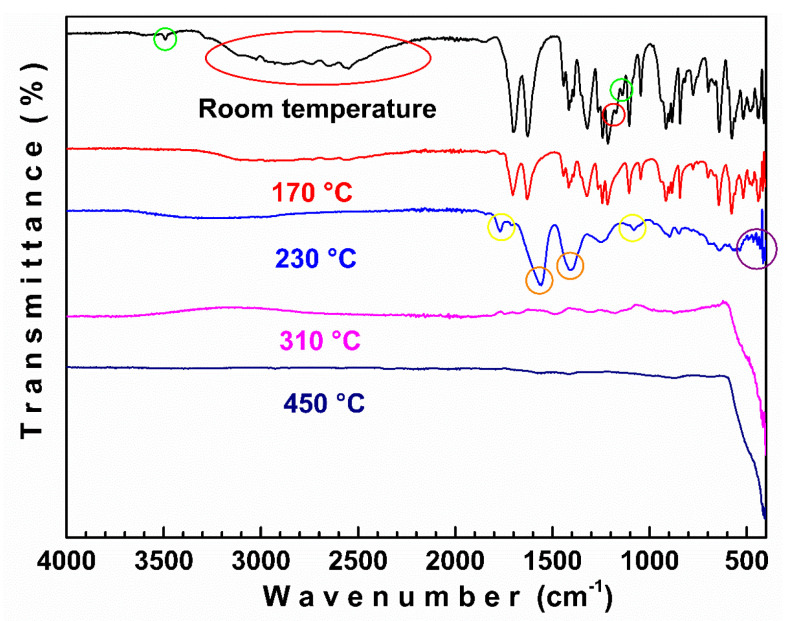
FTIR spectra of precursors and products of their decomposition at various temperatures for the ZnO polymeric precursor.

Figure 2 shows the FTIR spectra of the product decomposition sintered at 170, 230, 310, and 450 °C. As can be seen, initially (RT), the spectrum does not appear to contain crystallization water because the gel was previously dried at 120 °C for 6 h. In the spectrum, typical bands associated with CA and zinc citrate are observed [21,22,23,24].

The weak and sharp peak observed at 3490 cm^−1^ is typically associated with the hydrogen–oxygen stretch of the α–hydroxy group of the CA. The broad band from 2700 to 3100 cm^−1^ can be assigned to O–H and C–H bond stretching, which is indicative of the formation of a material with polymeric properties. The first weight loss (25 °C to 170 °C) can be ascribed to the elimination of absorbed or superficial water molecules. During this stage, the thermal degradation of the free CA and the citrate depolymerization take place. This is evidenced by the reduction in the intensities of the bands compared to that of the sample without thermal treatment (RT). The degradation of the free CA could be associated with the absence of the carbon–oxygen and hydrogen–oxygen stretch bands of the α-hydroxide (green circle) as a result of a dehydration reaction, presumably leading to the formation of free aconitic acid [22]. Additionally, the lower intensity of the band enclosed in the red circle may be related to the depolymerization of the zinc citrate chains.

The second weight loss (170 °C to 230 °C) shows an almost complete reduction in the intensity of the citrate bands. Two strong bands appear at 1560 cm^−1^ and 1446 cm^−1^ (orange circle), indicating the partial decomposition of zinc citrate, forming zinc acetate, Zn(CH_3_COO)_2_ [25]. This is confirmed by the reduction in the Zn–O stretching vibration in the fingerprint region (purple circle). On the other hand, the appearance of two additional bands located at 1770 cm^−1^ and 1080 cm^−1^ (yellow circle) confirms the conversion of free aconitic acid to free itaconic anhydride [22]. The next stage of weight loss (230–315 °C) could be attributed to the thermal decomposition of zinc acetate, which forms ZnO, and the decomposition of free itaconic anhydride [22]. The fourth stage, in the range of 315 °C to 370 °C, could be ascribed to the degradation of CA decomposition subproducts through a strongly exothermic combustion–oxidation process during which the total crystallization of ZnO takes place. Finally, in the last stage (370–450 °C), a small amount of any residual carbonaceous material is eliminated. By comparing the TGA/DTG curves of the Co–doped ZnO precursor with those of pure ZnO (Figure 1b), an additional peak is observed in the third stage, and the fourth stage of thermal decomposition occurs at a higher temperature than in pure ZnO. These differences could be associated with the formation of different metallic citrate molecules (Zn and Co), indicating the effect of doping incorporation in the polymeric network.

### 3.2. FTIR-ATR

**Figure 3:**
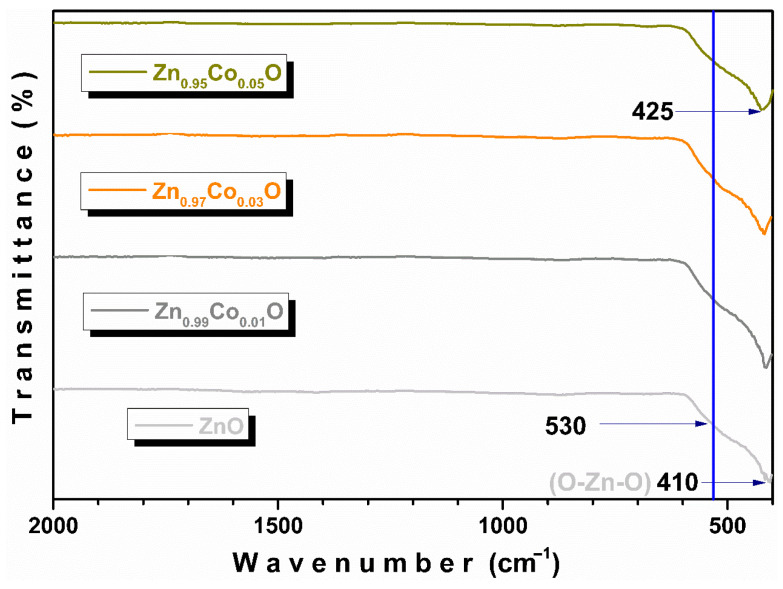
FTIR-ATR spectra of Zn_1−x_Co_x_O (0.00 ≤ x ≤ 0.05) samples.

Figure 3 displays the FTIR-ATR spectra of Zn_1−x_CoxO samples with x = 0.00, 0.01, 0.03, and 0.05. In the spectrum of the pure ZnO sample, two strong bands are observed. The first, centered around 410 cm^−1^, is attributed to antisymmetric stretching vibration modes of O–Zn–O bonds in tetrahedral coordination into a wurtzite crystalline structure [26]. The second one, around 530 cm^−1^, may be attributed to oxygen deficiency and/or vacancies in ZnO [27,28].

From the figure, it is observed that with the increase in doping concentration, the O–Zn–O mode at 410 cm^−1^ systematically shifts to a higher wavenumber, indicating the incorporation and/or replacement of Co ions into the ZnO structure, thereby modifying the O–Zn–O bonds. In fact, if cobalt substitutes zinc at its lattice sites, the vibrational modes must shift toward higher wavenumber regions, given that the atomic mass of Co (58.93 u.m.a) is lower than that of Zn (65.38 u.m.a). On the other hand, the band associated with oxygen deficiency does not change appreciably in intensity and position (blue line), indicating that the vacancies are probably bulk defects [29].

### 3.3. SEM Micrographs

**Figure 4:**
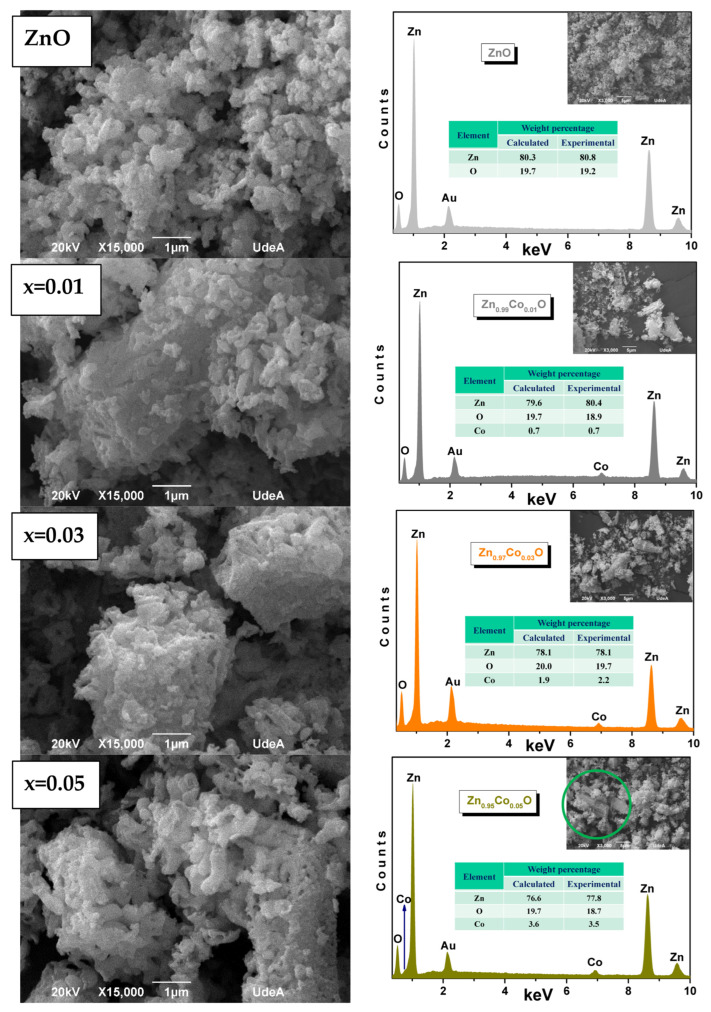
The left part shows SEM micrographs of pure ZnO and Zn_1−x_Co_x_O samples with x = 0.01, 0.03, and 0.05. The right part shows EDS spectra to 3000 X (upper insets) for each of the samples. The green circle in the Zn_0.95_Co_0.05_O sample indicates a morphology change compared to the ZnO sample.

SEM micrographs (15,000 X–1 μm) of Zn_1−x_Co_x_O with x = 0.00, 0.01, 0.03, and 0.05 samples are shown in the left part of Figure 4.

The image of the pure ZnO sample reveals that the crystals are constituted by agglomerated particles, irregular in shape and size with a wide distribution, indicating the presence of nanometric particles. As the cobalt concentration increases, no appreciable change in the particle’s morphology is observed; however, a larger particle size is evident in the Zn_0.95_ Co_0.05_ O sample [30]. Additionally, the micrographs show that as the Co concentration increases, the samples tend to exhibit a rougher surface and a more complex texture. This makes the system a promising candidate for applications, mainly in gas adsorption and/or photocatalytic performance [30]. The EDS spectra (right part of Figure 3), taken over the selected scan area (3000 X–5 um, see upper insets), evidences only the existence of Zn and O atoms in the pure ZnO and Zn and Co and O in the Zn_1−x_Co_x_O system, ruling out the presence of impurities in the samples. The experimental and calculated elemental compositions are within the limit of experimental error (see Tables). When the SEM micrographs are detailed to 3000 X, a new morphology (sheet–like, see green circle) appears in the sample with a higher Co content compared to the undoped ZnO, evidencing the effect of the amount of doping on the morphology of the system.

### 3.4. XRD

**Figure 5:**
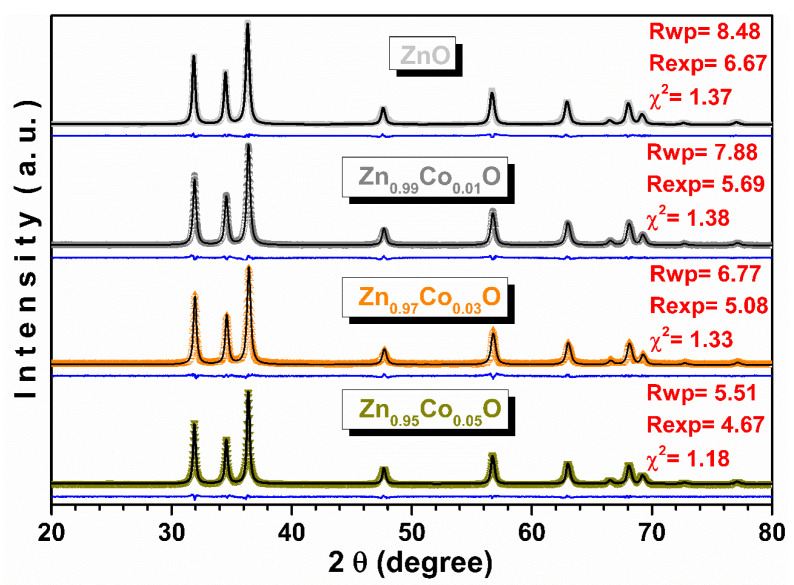
Rietveld analysis of XRD patterns for undoped and Co–doped ZnO samples. Semisolid symbols are experimental data, whereas black solid lines represent the fit. The blue lines below represent the difference pattern. The Rwp and Rexp factors along with χ^2^ are included.

**Figure 6:**
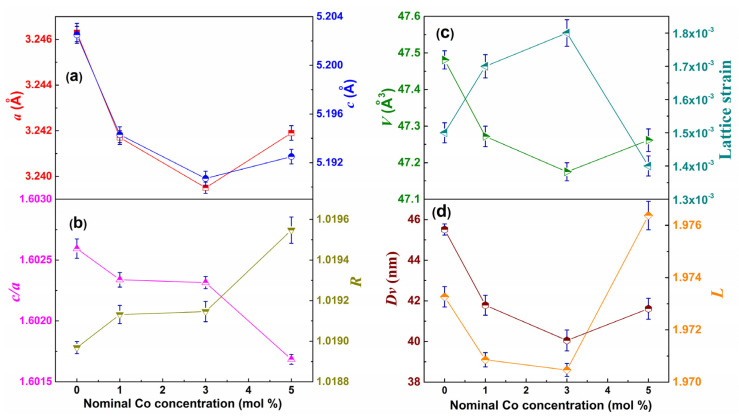
Variation in (**a**) *a* and *c* lattice parameters, (**b**) *c*/*a* ratio and lattice distortion degree (*R*), (**c**) volume and lattice strain, and (**d**) average crystallite size (*Dv*) and Zn–O bond length (*L*) parallel to the *c*–axis as a function of the nominal Co molar content. For the *a*, *c*, and *Dv* parameters, error bars are standard deviations of the fit, while the error bars of the other parameters are standard deviations of the results.

The refined diffraction patterns for all samples using the Rietveld method are presented in Figure 5. The obtained parameters of the fits along with the *R* (distortion degree), cell volume, lattice strain, *Dv*, and *L* (Zn–O bond length along the *c* direction) parameters are displayed in Figure 6. The Rwp and Rexp factors, as well as the χ^2^ (the goodness of fit) values, demonstrate the high quality of the refinement.

Only reflections belonging to the hexagonal–structure–type wurtzite with a P6_3_*mc* space group (JCPDS card no. 36–451) [31] were identified. Bragg peaks associated with other ZnO polymorphs [32] or Co CoO, Co_3_O_4_, or ZnCo_2_O_4_ phases were not evidenced within the technique’s detection limit [33,34]. From Figure 6, it is observed that the *a* and *c* parameters of Zn_1−x_Co_x_O gradually decrease until x = 0.03 and then increase when x reaches 0.05. The changes in the cell parameters observed in this study are very similar to those reported by Jabbar [10] and Hays et al. [35] in Zn_1−x_Co_x_O nanoparticles.

The ionic radii of high–spin tetrahedral Co^2+^ (0.58 Å) are slightly smaller than those of Zn^2+^ (0.60 Å) in a tetrahedral environment [36]. The decrease in lattice parameters for x = 0.03 could be related to the substitutional Co^2+^ ions occupying tetrahedral Zn^2+^ lattice sites in the wurtzite structure. The observed increase in the lattice parameters for x = 0.05 suggests the additional incorporation of Co^2+^ in octahedral coordination (interstitial position) and/or the presence of Co^3+^ ions with significant lattice defects. These observations are also evidenced by the changes in the *c*/*a*, *R*, and *L* parameters (see Figure 6). The lattice strain (ε) values [8] for the Zn_1−x_Co_x_O samples were (1.5, 1.7, 1.8, and 1.4) × 10^−3^ for x = 0.00, 0.01, 0.03, and 0.05, respectively. It is noted that with an increase in concentration, ε first increases and then decreases, similar to *L* and the lattice parameters, and inversely to the volume. In both the pure ZnO and Co–doped ZnO samples, the *c*/*a* parameter is in the range of 1.6017–1.6026, which is smaller than the *c*/*a* ratio for an ideal wurtzite structure (1.633). This may indicate the presence of oxygen vacancies [10] as also detected by FTIR. Figure 6 shows that the *Dv* decreases with an increase in cobalt content up to 3.0 molar %; thereafter, the value increases with a further increment of Co content up to 5.0 mol %. These results could be related to the fact that at a lower doping concentration, the Co ions modify the nucleation rate, inhibiting further growth of the ZnO crystal, while a higher concentration encourages crystallite growth. This observation is in good agreement with the SEM results.

### 3.5. Optical Properties

**Figure 7:**
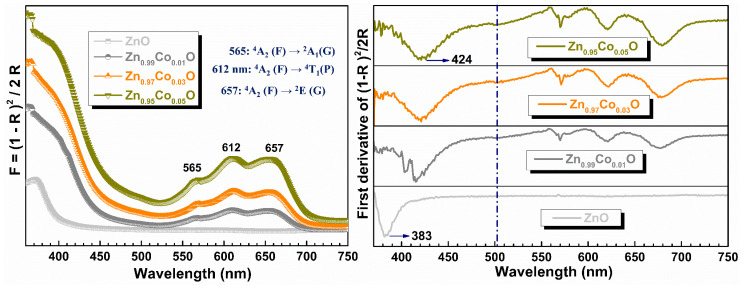
(**Left part**): optical absorbance diffuse reflectance spectra of ZnO and Zn_1−x_Co_x_O samples. The figure shows the d–d transitions of tetrahedrally coordinated Co^2+^ ions. (**Right part**): first derivative of absorbance spectra with respect to wavelength for Zn_1−x_Co_x_O samples.

The RT optical absorption spectra of ZnO and Co–doped ZnO nanoparticles with different concentrations are shown in the left part of Figure 7. In the pure ZnO sample, the absorption peak is centered around 375 nm. A gradual and systematic blue shift is observed in the Zn_1−x_Co_x_O samples as x increases with respect to the pure ZnO sample. This blue shift behavior could indicate the replacement of Co^2+^ ions at Zn^2+^ into the ZnO structure [8]. In the spectra of the Co–doped ZnO samples, the presence of three humps located at 565, 612, and 657 cm^−1^ is evident when compared to the undoped ZnO sample.

These peaks are ascribed to crystalline field splitting, resulting from d–d transitions of high–spin Co^2+^ ions (3d^7^, S = 3/2)) in a tetrahedral crystal symmetry, substituting Zn^2+^ sites within the ZnO hexagonal wurtzite structure [15,16,17]. These dd transitions from the ground state to their respective excited states are indicated in the figure. It is noticeable that the relative area under these three peaks gradually increases with the doping level, indicating higher absorption by Co^2+^ ions. Two additional humps, located at ~390 and ~495 nm, are observed for all Co–doped ZnO samples. The band around 390 nm is attributed to sub–band gap charge–transfer (CT) transitions, where Co^2+^ acts as the CT acceptor and the valence band serves as the CT donor [37]. The band at 495 nm is assigned to the ligand field transitions of Co^2+^ ions in a pseudo–octahedral environment [38], primarily on the particle surface. The notable increase in the absorption band due to the pseudo–octahedral Co^2+^ ions in the sample with x = 0.05 could explain the increase in the *a* and *c* lattice parameters and the morphology change at this doping molar concentration. The higher presence of these Co^2+^ ions increases the surface charge density, which could modify the crystal’s growth in a preferential orientation. The first derivative with respect to the wavelength of all synthetized samples is displayed in the right part of Figure 7. In this figure, Co^2+^ ions in a pseudo–octahedral environment are more readily observed. As x increases from 0.00 to 0.01, the peak centered at 383 nm, corresponding to the band edge, suddenly disappears. When these results are compared with the first derivative with respect to the wavelength of Fe–doped ZnO nanoparticles [39], it is observed that Co^2+^ ions have a more pronounced effect on altering the absorption edge. This fact may lead to the development of theoretical work, using first–principles calculations, that attempts to explain these results.

**Figure 8:**
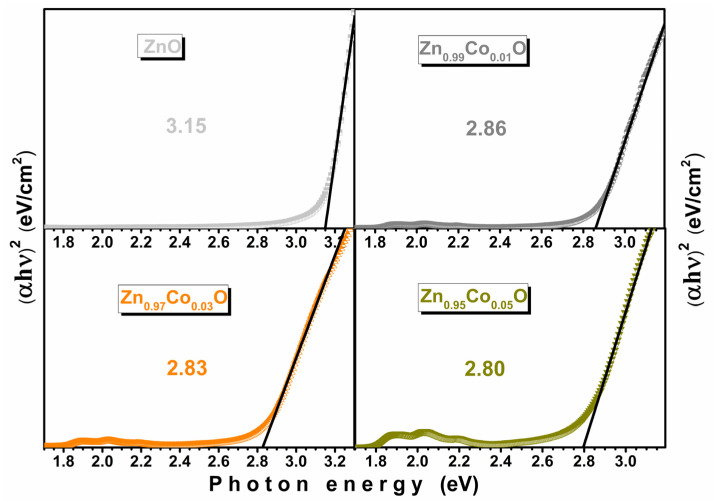
Variation in the calculated band gap according to the Kubelka–Munk equation for Zn_1−x_Co_x_O samples with x = 0.00, 0.01, 0.03, and 0.05.

The optical direct band gap (Eg) energies for the synthesized samples were determined using the Kubelka–Munk equation [15,40]. These results are shown in Figure 8. The Eg value of the ZnO sample was found to be 3.15 eV, which is similar to previously reported results [15,16]. The observed decrease in the Eg with increasing doping concentration cannot be associated with the quantum confinement effect. This is because the crystallite sizes observed in this work are larger than the exciton Bohr radius of the ZnO [41]. Additionally, based on our results, variation in the crystallite size and lattice parameters can also be ruled out. Therefore, structural changes caused by the dopant incorporation play a dominant role in the decrease in the forbidden band. Stronger exchange interactions between the localized d electrons of the Co^2+^ ions substituting for Zn^2+^ ions and the s and p electrons of the ZnO host band could be responsible for the band gap shrinkage [15].

### 3.6. Magnetic Properties

**Figure 9:**
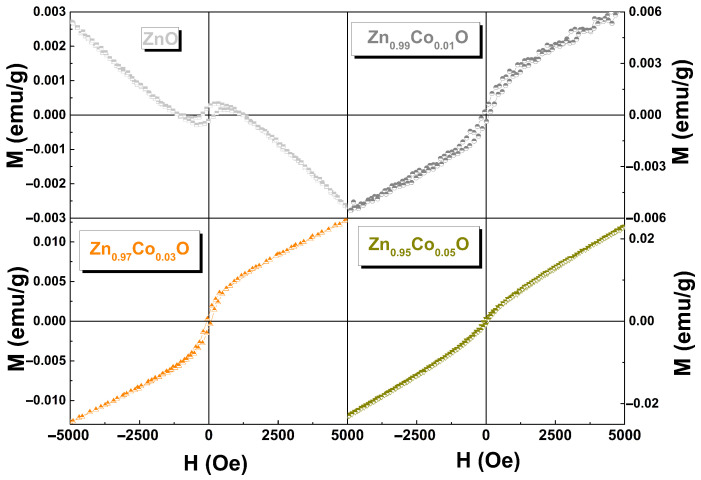
RT hysteresis loops for Zn_1−x_Co_x_O samples with x = 0.00, 0.01, 0.03, and 0.05.

M vs. H curves at RT for Zn_1−x_Co_x_O with x = 0.00, 0.01, 0.03, and 0.05 are shown in Figure 9. In the pure ZnO sample, a mixture of a diamagnetic (DM) component along with a clear hysteresis loop is observed, evidencing room temperature ferromagnetism (RTFM). This detected behavior is not surprising according to previous reports [42,43].

The weak FM behavior in undoped ZnO has generally been associated with oxygen and/or zinc vacancies at or near the surface, a wide size particle distribution, grain boundaries, uncompensated spin systems on the surface of the nanoparticles, and/or lattice imperfections occasioned by the stress [9]. In Co–doped ZnO samples, the M vs. H curves at RT consist of an open curve, indicating an FM contribution, superimposed with a linear part due to a paramagnetic (PM) contribution. In these samples, the coercive field (Hc) decreases with increasing Co content. This behavior has also been reported by other authors [16,44]. It can be seen that the PM component increases with increasing x, and there is no sign of saturation at higher applied magnetic fields. Such behavior could indicate the presence of isolated Co^2+^ ions that are not participating in the magnetic ordering. These data support a weak RT ferromagnetism (RTFM) in these samples. It is worth mentioning that, according to our experimental results, the weak FM observed in the samples is not due to any secondary phase or clusters.

**Figure 10:**
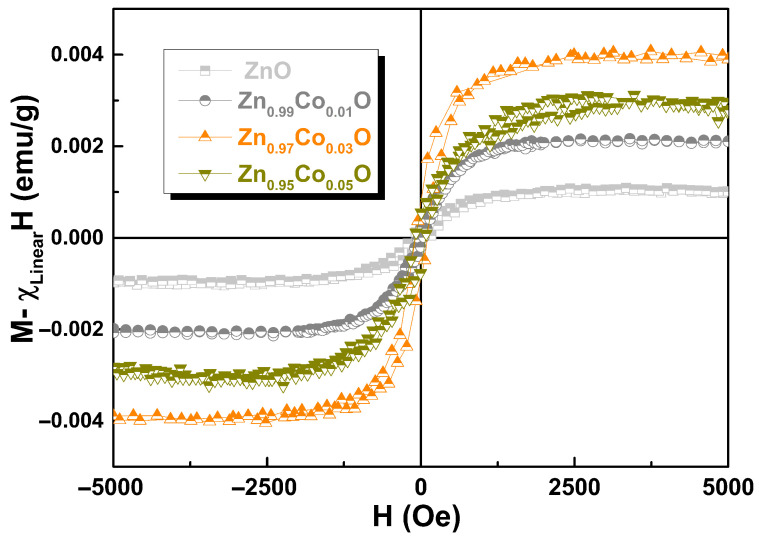
RT hysteresis loops for Zn_1−x_Co_x_O samples with x = 0.00, 0.01, 0.03, and 0.05, where the linear component has been subtracted.

**Figure 11:**
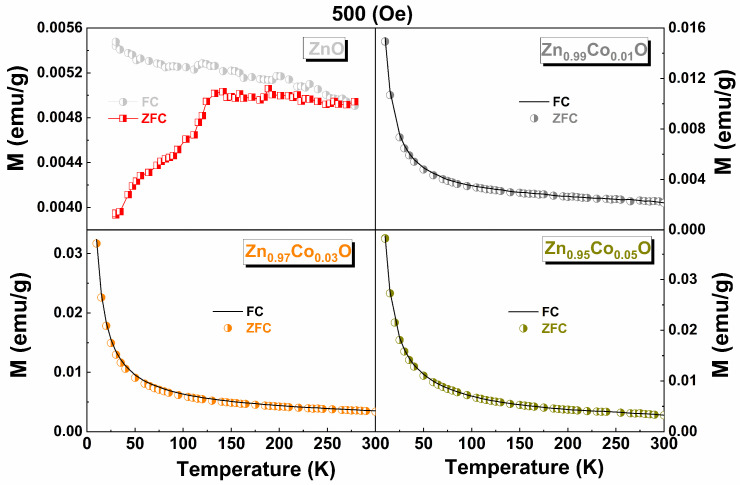
FC and ZFC curves for Zn_1−x_Co_x_O samples with x = 0.0, 0.01, 0.03, and 0.05. The samples with x = 0.01, 0.03, and 0.05 were fitting using the modified Curie−Weiss law (see text).

Figure 10 shows the RT M vs. H curves for Zn_1−x_Co_x_O (0.00 ≤ x ≤ 0.05) samples. The linear component (χ) has been subtracted (diamagnetic for ZnO and paramagnetic for Co–doped ZnO samples) to illustrate the actual Ms from the FM phase. We observe that the Ms value first increases and then decreases with increasing Co doping, exhibiting the highest Ms value for the Zn_0.97_Co_0.03_O sample, as reported by [8]. The zero–field–cooling (ZFC) and field–cooling (FC) curves for the undoped ZnO and Co–doped ZnO samples are shown in Figure 11. The pure ZnO sample shows branching between ZFC and FC, which is characteristic of superparamagnetic (SPM) behavior, with a blocked state in the low–temperature regime around 135 K. Additionally, the ZFC curve exhibits an additional peak around 50 K. This discrepancy in the M vs. H curve could be attributed to finite particle size with high agglomeration, lattice imperfections (microstrain), and/or the presence of uncompensated spin systems on the nanoparticle surfaces [45]. For the Zn_0.99_Co_0.01_O sample, a small difference is observed between the ZFC and FC curves, which increases for the Zn_0.97_ Co_0.03_ O sample and then decreases for the Zn_0.97_ Co_0.05_ O sample. The presence of such irreversibility should not exist in a purely paramagnetic system; rather, it indicates the existence of weak ferromagnetic behavior at RT [44].

These curves were fitted using the modified Curie–Weiss law, χ = χ_0_ + C/ (T + θ) [46], where χ_0_ represents non–PM contributions, C = Nμ^2^/3K_B_ (N is the number of magnetic ions per g, μ = is the magnetic moment of the ion, and K_B_ is the Boltzmann constant), and θ is the Curie–Weiss temperature. The results of these fits are shown in Table 1. These fits revealed an increase in C as x increases, confirming the progressive doping of Co^2+^ ions. Only positive values were obtained for θ, indicating that the interactions between Co^2+^ ions are mainly of antiferromagnetic (AFM) character.

As Co doping increases, θ also increases, indicating a progressive increment in the AFM contribution. It is also found that χ_0_ increases up to x = 0.03 and then decreases for x = 0.05, which is in full agreement with the results presented in Figure 10.

According to our results, it is evident that Co^2+^ ions modify the magnetic behavior of pure ZnO. The higher Ms observed in Zn_1−x_Co_x_O samples with x= 0.01 and 0.03 compared to undoped ZnO might be correlated with the perturbation/alteration and/or changes in the electronic structure of ZnO. This could be occasioned by the exchange interaction between the localized magnetic dipole moments of the Co^2+^ ions and the free delocalized charge carriers from the valence band. The decrease in the weak FM signal in Zn_0.95_Co_0.05_O has been associated with an increase in AFM–coupled spins. It is worth mentioning that the Ms values in the Co–doped ZnO samples show similar behavior as the cell, L, and ε parameters. Therefore, lattice imperfections caused by stress must not be ruled out when accounting for the weak FM signal observed in these samples.

## 4. Conclusions

We have experimentally investigated the crystallographic, vibrational, morphological, optical, electronic, and magnetic properties of undoped and Co–doped ZnO samples. These samples were synthesized via a simple and cost–effective method characterized by few handling steps. We analyzed the thermal decomposition of the ZnO polymeric precursor using FTIR spectroscopy, in which Zn(CH_3_COO)_2_ was identified as the main intermediate product. The samples were free from contamination. The results from XRD demonstrated that all samples exhibited the wurtzite ZnO phase with an average crystallite size in the nanometric range. SEM micrographs show that with increasing cobalt concentration, there is a gradual increase in the surface roughness of the samples, and a new morphology (sheet–like) is observed for x = 0.05. FTIR-ATR and UV–vis absorbance spectra displayed a blue shift with increasing Co doping, which evidenced the incorporation and/or substitution of Co^2+^ ions in the host semiconductor. The calculated band gap energy decreased as the Co molar concentration increased. Only high–spin Co^2+^ ions were detected, located in different environments: tetrahedral core sites and pseudo–octahedral surface sites. Undoped and Co–doped ZnO samples showed weak RTFM, where the 3 mol % Co–doped ZnO sample displayed the maximum Ms and the Hc decreased with increasing Co content. For pure ZnO, this behavior was associated with oxygen vacancies, finite particle size, a wide particle size distribution, and uncompensated spin systems on the surface. The reduction in the weak FM signal after a molar concentration of 3% Co is likely due to increased antiferromagnetic coupling.

## Figures and Tables

**Table 1:** Parameters for Zn_1−x_Co_x_O samples as a function of x from the modified Curie–Weiss law fit of magnetization of non–paramagnetic contributions (Mo), Curie constant (C), and Curie–Weiss temperature (θ).

x in Zn_1−x_Co_x_O	Mo (emu/g)	C (emu K/g Oe)	θ (K)
0.01	0.94	0.15	1.26
0.03	1.29	0.33	1.41
0.05	1.09	0.45	2.72

## Data Availability

The original contributions presented in this study are included in the article. Further inquiries can be directed to the corresponding author.
